# Clinicopathologic features and abnormal signaling pathways in plasmablastic lymphoma: a multicenter study in China

**DOI:** 10.1186/s12916-022-02683-9

**Published:** 2022-12-15

**Authors:** Di Shi, Lin Gao, Xiao-Chun Wan, Jin Li, Tian Tian, Jue Hu, Qun-Ling Zhang, Yi-Fan Su, Yu-Peng Zeng, Zi-Juan Hu, Bao-Hua Yu, Xiao-Qiu Li, Ping Wei, Ji-Wei Li, Xiao-Yan Zhou

**Affiliations:** 1 Department of Pathology, Fudan University Shanghai Cancer Center, Shanghai, 200032 China; 2 Department of Oncology, Shanghai Medical College, Fudan University, Shanghai, 200032 China; 3 Institute of Pathology, Fudan University, Shanghai, 200032 China; 4 GenePlus-Shenzhen, Shenzhen, 518000 People’s Republic of China; 5 Institute of Microbiology, Chinese Academy of Sciences, Beijing, 102199 China; 6 Department of Oncology, Hunan Cancer Hospital, Changsha, 410000 China; 7 Department of Oncology, The Second Xiangya Hospital, Central South University, Changsha, China

**Keywords:** Plasmablastic lymphoma, Immunocompetent, RNA-sequencing

## Abstract

**Background:**

Plasmablastic lymphoma (PBL) is a rare but aggressive B-cell lymphoma subtype with poor prognosis. Knowledge about the etiology, clinicopathologic and molecular features, and outcomes of PBL is limited. This study aimed to examine the clinicopathologic characteristics, therapeutic approaches, and clinical outcomes of PBL patients in a Chinese population.

**Methods:**

A total of 102 PBL patients were recruited from three cancer centers. The pathologic features and clinical outcomes of 56 patients with available treatment details and follow-up data were reviewed and analyzed. RNA sequencing was performed in 6 PBL and 11 diffuse large B-cell lymphoma (DLBCL) patients.

**Results:**

Most patients in our cohort were male (*n* = 36, 64.3%), and 35 patients presented with Ann Arbor stage I/II disease at diagnosis. All these patients showed negative findings for human immunodeficiency virus, and the vast majority of patients in our cohort were immunocompetent. Lymph nodes (*n* = 13, 23.2%) and gastrointestinal tract (*n* = 10, 17.9%) were the most commonly involved site at presentation. Post-treatment complete remission (CR) was the only prognostic factor affecting overall survival (OS) and progression-free survival (PFS) in the multivariate analysis. RNA-seq demonstrated that B-cell receptor (BCR), T-cell receptor (TCR), P53, calcium signaling, and Wnt signaling pathways were significantly downregulated in PBLs compared with GCB (or non-GCB) DLBCLs.

**Conclusions:**

In this multicenter study in the Chinese population, PBL mainly occurred in immunocompetent individuals and most patients present with early-stage disease at diagnosis. Post-treatment CR was an important prognostic factor affecting OS and PFS. RNA-seq showed that the B-cell receptor (BCR), P53, calcium signaling, cell adhesion molecules, and Wnt signaling pathways significantly differed between PBL and GCB (or non-GCB) DLBCL, which provided theoretical basis for its pathogenesis and future treatment.

**Supplementary Information:**

The online version contains supplementary material available at 10.1186/s12916-022-02683-9.

## Background

Plasmablastic lymphoma (PBL) is a distinct and rare subtype of B-cell lymphoma that exhibits a plasmablastic morphology but shows a plasma cell-like immunophenotype [1, 2]. Previous studies have reported that PBL predominantly occurs in immunocompromised individuals, such as those with human immunodeficiency virus (HIV) infection, organ transplantation, and autoimmune diseases [1, 3]. Frequent Epstein-Barr virus (EBV) infection and *MYC* gene aberrations present in PBL patients are reported as adverse prognostic factors and may contribute to lymphomagenesis in these patients [4]. However, the exact pathogenesis of PBL remains largely unknown and requires further investigation.

Diagnosing PBL is sometimes challenging, as it shares some similar clinicopathological features with myeloma and diffuse large B-cell lymphoma (DLBCL). Although comparative genomic hybridization analysis has revealed that the genomic aberration profile of PBL seems to be more similar to that of DLBCL than plasma cell myeloma (PCM) [5], the prognosis of PBL was reported to be significantly worse than to that of DLBCL [6], with an estimated 2-year overall survival (OS) of < 50% [7, 8]. Therefore, a better understanding of the biology and pathophysiology of PBL may help in improving the survival outcomes.

As most studies on PBL were case reports and small case series, the pathogenesis, standard treatment approaches, and prognostic factors remain largely unknown. Furthermore, clinicopathological features and survival outcomes may vary across populations. Therefore, this study aimed to analyze the clinicopathologic characteristics, therapeutic approaches, and clinical outcomes of PBL patients in a Chinese population. In addition, RNA-sequencing was performed to identify the differences between PBL and DLBCL.

## Methods

### Patient group

All the patients diagnosed with PBL between January 2008 and October 2019 at the Fudan University Shanghai Cancer Center, Hunan Cancer Hospital, and the Second Xiangya Hospital were examined in this study. Most patients were referred to our institution for consultation after a biopsy was performed. Clinical data such as patients’ age, sex, HIV status, medical history, primary tumor site, Ann Arbor stage, therapies, and clinical outcomes were obtained from medical records. This study was performed in accordance with the Declaration of Helsinki and approved by the ethics committee of each participating medical center. All participants provided written informed consent.

The main immunohistochemical markers (CD20, CD79a, PAX-5, CD30, CD38, CD138, MUM1, and Ki67) were reviewed by two pathologists (Wan and Yu).

### Statistical analysis

Progression-free survival (PFS) was calculated from the date of diagnosis to the date of disease progression, relapse, or death by any cause. OS was defined as the time from the date of initial diagnosis to the date of death or last contact. Using univariate analysis, the prognostic role of patient age, sex, disease stage, B symptoms, increased serum lactate dehydrogenase (LDH) level, Ki67, EBER, and patient complete remission (CR) status was evaluated. Prognostic factors (*p* < 0.05) were further examined using multivariate analysis with Cox regression. PFS and OS were estimated using the Kaplan–Meier method. Survival curves were compared using the log-rank test. Chi-square tests or Fisher’s precision probability tests was used to analyze the clinical differences in different patient groups. Statistical analysis was performed using GraphPad Prism (version 5, GraphPad Software) and R (version 3.5.1, R Foundation for Statistical Computing, Vienna, Austria). Statistical significance was set at *P* < 0.05.

### *Immunohistochemistry (IHC) and *in situ* hybridization for EBV-encoded RNA*

Hematoxylin and eosin staining and *IHC studies* were performed on formalin-fixed and paraffin-embedded tissue sections using standard methods. Primary antibodies against CD20, CD79a, PAX-5, CD38, CD138, and MUM1 (Ventana Medical Systems, USA) were applied on a BenchMark XT automated immunostainer (Ventana Medical Systems) with Cell Conditioning heat retrieval solution (Ventana Medical Systems). Appropriate internal controls (lymphocytes) and external controls (tonsils) were also included in each section. The morphology and IHC results were reviewed by two pathologists (Wan and Yu).

Detection of EBV-encoded small RNA (EBER)-1/2 was performed with proper controls using an ISH kit (Triplex International Bioscience, China) following the manufacturer’s instructions.

### RNA-sequencing

RNA was extracted using the AllPrep Kit (Qiagen). Sequencing libraries for RNA-sequencing were prepared using TruSeq RNA Library Prep Kit V2 (Illumina). Paired-end 100 bp read sequencing was performed on a HiSeq 2500 system using Illumina TruSeq V3 chemistry. Paired-end reads were mapped to the human genome (NCBI build 37) by the gapped aligner STAR 2.4.117, using the two-pass method and parameters recommended by the NCI Genomic Data Commons (GDC) 18. The alignment file was used to calculate the raw digital gene expression values by HTseqcount software 0.7.219, using the intersection-nonempty model, which were further analyzed to provide digital gene expression values. The alignment file was also used for variant calling by VarScan2 with selection based on variant read count ≥ 3 and variant read frequency ≥ 0.1.

### Reverse-transcriptase quantitative PCR (RT-qPCR)

RT-qPCR was performed as previously described [9, 10]. RNA was extracted from paraffin-embedded tissue using the AllPrep Kit, according to the manufacturer’s instructions (Qiagen, San Diego, CA, USA). cDNA was synthesized with the Reverse Transcriptase Kit (Takara, # RR036A). Then, qPCR was performed using SYBR Green according to a standard protocol (Takara, #RR420A). Glyceraldehyde-3-phosphate dehydrogenase (GAPDH) served as internal control. The primer sequences are provided in Additional file 1: Table S1.

## Results

### Patient characteristics

A total of 102 patients were diagnosed with PBL at our three cancer centers and the clinical characteristics are summarized in Additional file 1: Table S2.Treatment details and follow-up data were available for 56 patients and the clinicopathologic parameters, treatment, and survival outcome of these patients have been listed in Table 1. Of the 56 patients, most patients were male (*n* = 36, 64.3%), with a median age of 55.0 (range 10–79) years. PBL occurred at various sites, with the lymph nodes (*n* = 13, 23.2%) and gastrointestinal tract (*n* = 10, 17.9%) being the most commonly involved locations, followed by the oral cavity (*n* = 9, 16.1%). Most patients (*n* = 35) presented with Ann Arbor stage I/II disease at diagnosis. Elevated LDH level (17/41, 41.5%) and B symptoms (22/49, 44.9%) were observed in less than half of the patients. All these patients showed negative findings for HIV, and immunosuppression (immune-related disease, post transplantation, and current or previous malignancy) was noted in only 4 patients [scleroderma (*n* = 2) and organ transplantation (*n* = 2)].

**Table 1:** Clinicopathologic characteristics of the 56 PBL patients with available follow-up data in our study

Variable	Total *N* (%)
Patients	56
Age (years)	
> 60	23 (41.1)
≤ 60	33 (58.9)
Median (years)	55.0 (range 10–79)
Sex	
Male	36 (64.3)
Female	20 (35.7)
Site(s) of involvement	
Lymph involvement	13 (23.2)
Gastro-intestinal tractus	10 (17.9)
Oral cavity	9 (16.1)
Bone	7 (12.5)
Soft tissue	4 (7.1)
Ear, nose, throat site	3 (5.4)
Maxillary sinus	2 (3.6)
Skin	3 (5.4)
Lung	1 (1.0)
Adrenal gland	1 (1.0)
Peritoneum	1 (1.0)
Liver	1 (1.0)
Pleura	1 (1.0)
Ann Arbor stage	
I or II	35 (62.5)
III or IV	21 (37.5)
Immunohistochemistry	
CD38	28/31 (90.3)
CD138	29/42 (69.0)
MUM1	42/44 (95.4)
EBER	10/20
Ki67(median)	80%
Treatment	
Surgery	4 (7.1)
Chemotherapy	
CHOP	43 (76.8)
DA-EPOCH	3 (5.4)
Bor-based chemotherapy	4 (7.1)
Other	4 (7.1)
Radiation therapy	2 (3.6)
Therapy response	
CR	25 (44.6)
PR	17 (30.4)
SD	1 (1.8)
PD	13 (23.2)
Status at last follow-up	
Dead	22 (39.3)
Alive	34 (60.7)

*Bor*, bortezomib; *CR*, complete remission; *PR*, partial remission; *SD*, stable disease; *PD*, progressive disease

**Fig. 1:**
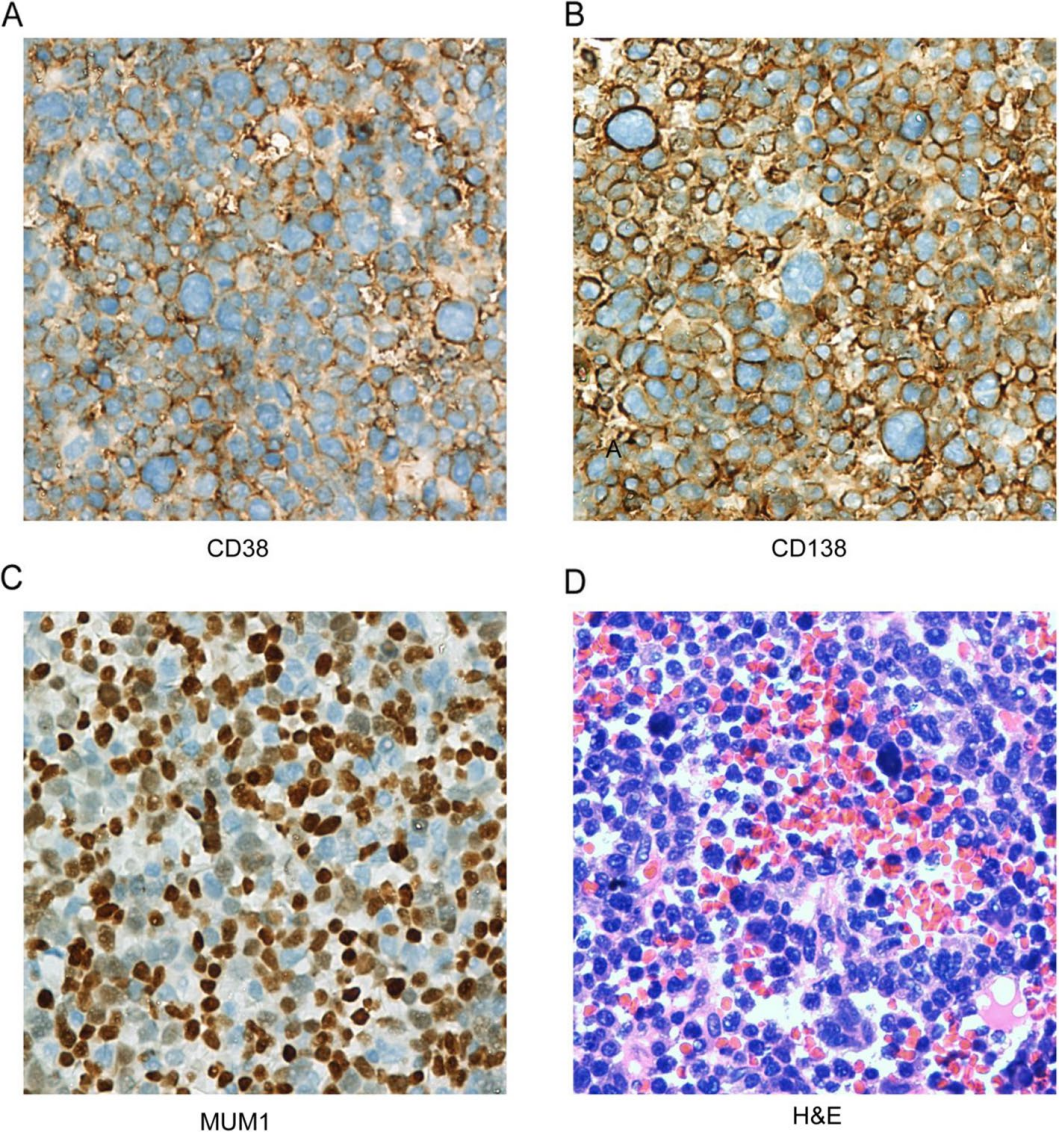
Representative case of plasmablastic lymphoma. **A–C** CD38, CD138, and MUM1 were positive by immunohistochemistry (× 400). **D** Neoplastic cells have plasmablastic morphology, with a prominent nucleolus and moderate amount of cytoplasm

Immunoblastic-like morphology and plasmacytic immunophenotypes were observed in patients with PBL (Fig. 1A–D). Plasma cell markers CD38 (Fig. 1A), CD138 (Fig. 1B), and MUM1 (Fig. 1C) were ubiquitously expressed in PBL patients, with positive rates of 90.3%, 69.0%, and 95.4%, respectively, for the entire cohort (Table 1). CD30 was positive in 23.8% of the tested patients (*n* = 42). All the patients showed negative findings for CD20, CD79a, and PAX-5. The Ki-67 expression was relatively high in our study, with a median value of 80% and a wide range between 40% and 100%. EBER expression was observed in 50% of the tested patients (*n* = 20). *MYC* expression was assessed in 14 patients, and 10 patients showed positive findings.

### Treatment

Treatment modalities in our cohort were as follows: 50 (89.3%) received chemotherapy alone, 2 received radiotherapy combined with chemotherapy, 2 (3.6%) received surgery and adjuvant chemotherapy, and 2 (3.6%) with localized disease (thyroidectomy for thyroid involvement and hemicolectomy for right hemi-colon involvement) received curative surgical resection alone. The chemotherapy regimens used are listed in Table 1. CHOP or CHOP-like chemotherapy was the most common regimen used in 43 patients. Bortezomib combined with chemotherapy, including PAD (bortezomib, doxorubicin, and dexamethasone), CHOP, and BADT (bortezomib, doxorubicin, dexamethasone, and lenalidomide), was used as first-line treatment in 4 patients. Other chemotherapy regimens, such as DA-EPOCH, GDP (gemcitabine, cisplatin, and dexamethasone), and GEMOX (gemcitabine and oxaliplatin), were used in seven cases.

### Response to treatment

In our cohort, 44.6% (*n* = 25) of the patients achieved CR after treatment, 30.4% (*n* = 17) achieved PR, 1.8% (*n* = 1) SD, and 23.2% (*n* = 13) showed PD. When combined with chemotherapy, bortezomib did not improve the CR rate compared to the CHOP group (*p* > 0.05). The two patients with localized disease achieved durable CR after surgery alone.

### Clinical outcome and prognosis parameters

**Fig. 2:**
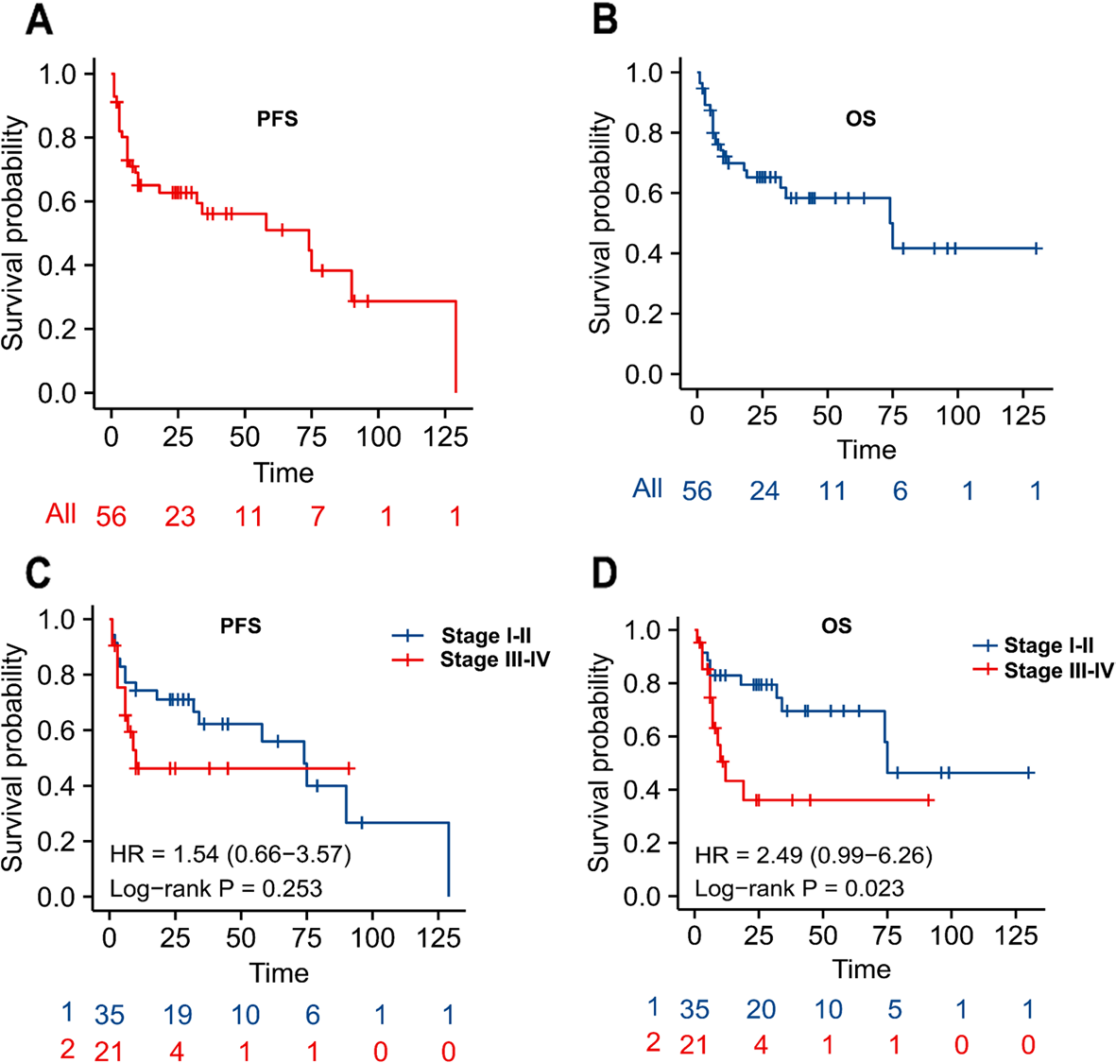
**A**, **B** Progression-free survival (PFS) and overall survival (OS) of all patients. **C**, **D** Comparison of progression-free survival (PFS) and overall survival (OS) between patients with stage I/II and III/IV disease

The median follow-up duration was 23.0 (range, 1–130) months. At the last follow-up, 22 (39.3%) PBL patients died. The 2-year PFS and OS rates in our cohort were 59.4% and 65.1%, respectively (Fig. 2A, B).

Prognostic factors determined by univariate and multivariate analyses are summarized in Table 2. Univariate analysis revealed that achievement of a CR (*p* = 0.001) was an adverse prognostic factor affecting PFS, which was not associated with other clinical parameters such as clinical stage (Fig. 2C). Clinical stage (*p* = 0.02) (Fig. 2D), serum LDH level (*p* = 0.03), and CR (*p* = 0.001) after treatment were important prognostic factors of OS, as revealed by univariate analysis. In the multivariate analysis, achievement of CR remained the only significant factor affecting OS and PFS.

**Table 2:** Univariate and multivariate analysis of prognostic factors for survivals (by Cox regression)

Clinical factor	Progression-free survival				Overall survival			
	Univariate		Multivariate		Univariate		Multivariate	
	*P*	HR (95%CI)	*P*	HR (95%CI)	*P*	HR (95%CI)	*P*	HR (95%CI)
Age (≥ 60y, *n* = 23 vs. < 60y, *n* = 33)	0.78	1.12 (0.51–2.47)			0.86	0.93 (0.39–2.17)		
Gender (male, *n* = 36 vs. female, *n* = 20)	0.82	0.91 (0.41–2.05)			0.12	1.93 (0.84–4.47)		
Primary site (oral, *n* = 9 vs. extra oral, *n* = 47)	0.67	0.82 (0.33–2.04)			0.25	0.57 (0.22–1.47)		
Ann Arbor stage (III/IV, *n* = 21 versus I/ II, *n* = 35)	0.27	1.58 (0.70–3.56)			0.03	2.58 (1.10–6.07)	0.66	1.32 (0.37–4.65)
B symptom (yes, *n* = 22 vs. no, *n* = 27)	0.36	0.65 (0.26–1.63)			0.38	0.66 (0.26–1.66)		
LDH level (elevated, *n* = 17 versus normal, *n* = 24)	0.16	0.51 (0.19–1.32)			0.03	0.33 (0.12–0.91)	0.06	0.35 (0.12–1.05)
EBER (positive, *n* = 10 vs. negative, *n* =10)	0.37	0.60 (0.19–1.85)			0.21	0.49 (0.16–1.49)		
Ki67 (≥ 80%, *n* = 40 vs. < 80%, *n* = 16)	0.34	0.63 (0.25–1.61)			0.56	0.75 (0.28–1.98)		
Complete response (yes, *n* = 25 versus. No, *n* = 31)	0.001	3.51 (1.65–7.50)	0.003	3.89 (1.60–9.45)	0.001	7.03 (2.33–21.17)	0.002	13.24 (2.58–67.99)

### RNA-sequencing results and validation by RT-qPCR

**Fig. 3:**
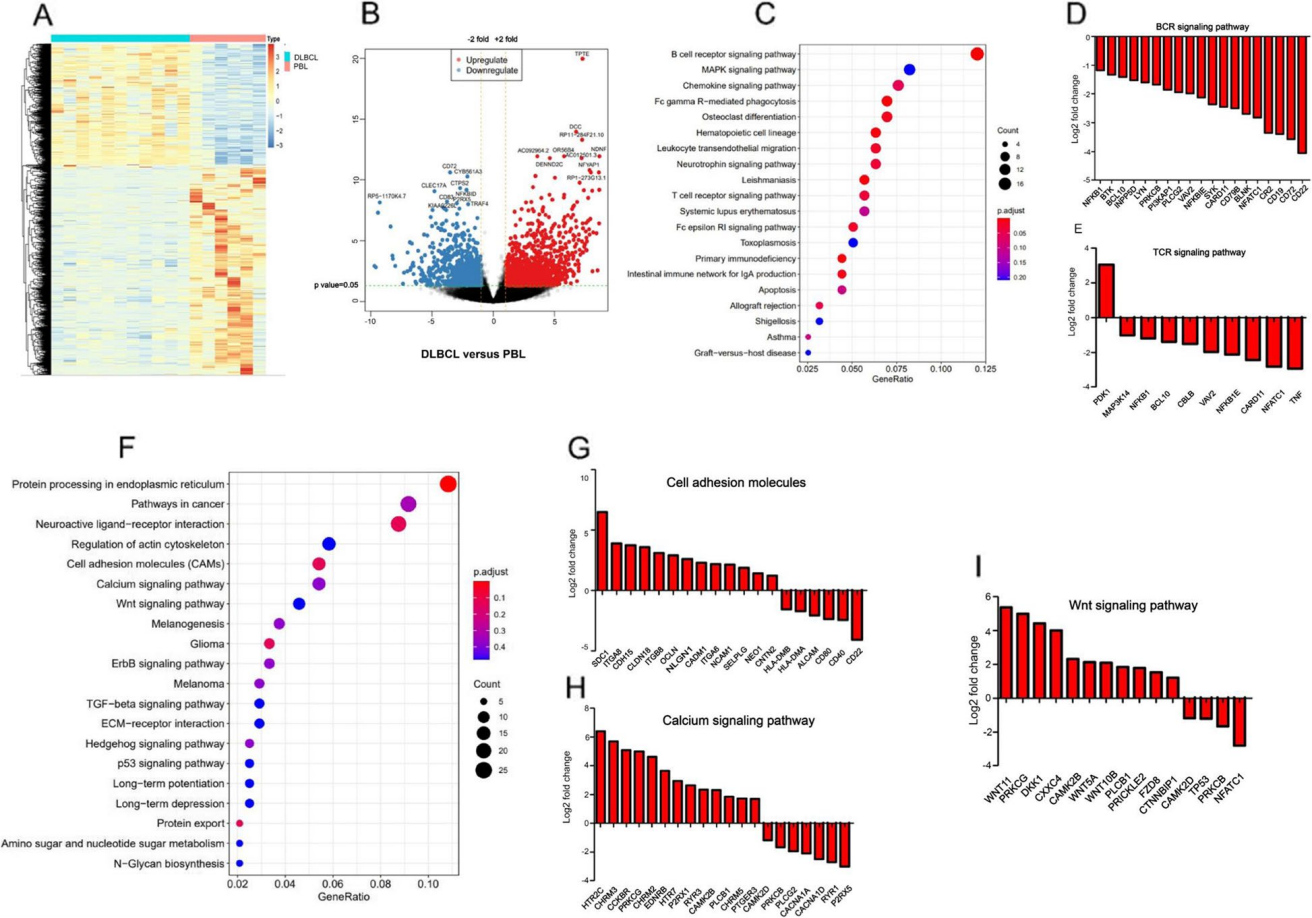
Expression profiles. **A** Hierarchical clustering and heat map of DEGs between PBL and DLBCL. Red blocks represent the overexpressed genes, while the blue blocks represent the lowly expressing genes. **B** Volcano plot of DEGs (FDR < 0.05) between PBL and DLBCL. **C** Significantly downregulated pathways in PBL compared to DLBCL. **D**, **E** Upregulation and downregulation of DEGs of BCR and TCR signaling pathways in PBLs compared to DLBCL. **F** Significantly upregulated pathways in PBL compared to DLBCL. **G–I** Upregulation and downregulation of DEGs of cell adhesion molecules, calcium, and Wnt signaling pathways in PBLs compared to DLBC

RNA-seq was performed in 6 PBL and 11 DLBCL cases. No difference was found between the PBL and DLBCL patients in gender, age, primary sites, and disease stage (Additional file 1: Table S3). As shown in Fig. 3A, a clustered heat map exhibits the profiling of the differentially expressed genes (DEGs) between PBL and DLBCL. Red blocks represent the overexpressed genes, while the blue blocks represent the lowly expressing genes. The heat map clustering showed that the gene expression pattern of PBL was distinguished from DLBCL. Compared with DLBCL, a total of 1507 DEGs were identified in the PBL group, including 1049 upregulated and 458 downregulated genes. The upregulated or downregulated genes are represented in a volcano plot (Fig. 3B).

Biological pathways and themes underlying the malignancy-specific gene expression patterns were identified. KEGG showed that some important pathways were downregulated in PBL compared to DLBCL, including BCR and TCR signaling pathways (Fig. 3C). Many BCR signaling pathway genes were significantly (FDR = 0.001, hypergeometric test) expressed at lower levels in PBLs than in DLBCLs. Individual BCR signaling genes (*CD22*, *CD72*, *CD19*, *CR2*, *NFATC1*, *BLNK*, *CD79N*, *CARD11*, *SYK*, *NFKBIE*, and *VAV2*) and TCR signaling genes were repressed by two-fold or more on average in PBLs (Fig. 3D, E). Compared with that noted in DLBCL, many biological pathways were upregulated in PBL, such as cell adhesion molecules, calcium, and Wnt signaling pathways (Fig. 3F–I). Cell adhesion genes (*SDC1*, *ITGA8*, *CDH15*, *CLDN18*, *ITGB8*, *OCLN*, and *NLGN1*) were higher in PBLs.

The gene expression profiles of the germinal center subtype (GCB) and non-germinal center subtype (non-GCB) DLBCL were further analyzed, retrospectively. The heat map clustering and volcano plot demonstrated that the gene profiling was different between PBL and GCB-DLBCL (Additional file 1: Fig. S1, A-B). Compared with GCB-DLBCL, no elevated important pathways in PBL were observed by KEGG, while BCR and P53 signaling pathway was significantly downregulated in PBL (Additional file 1: Fig. S1, C-F). The profiling of the different expressed genes and pathways between non-GCB DLBCL and PBL were also identified (Additional file 1: Fig. S2, A-B). KEGG showed that some important biological pathways were significantly upregulated (Additional file 1: Fig. S2, C) and downregulated (Additional file 1: Fig. S2, D) in PBL compared to non-GCB-DLBCL. BCR, TCR, Jak-Stat, and TLR signaling pathways were significantly downregulated in PBL and the detailed downregulated genes were shown in Additional file 1: Fig. S2(E–H).

**Fig. 4:**
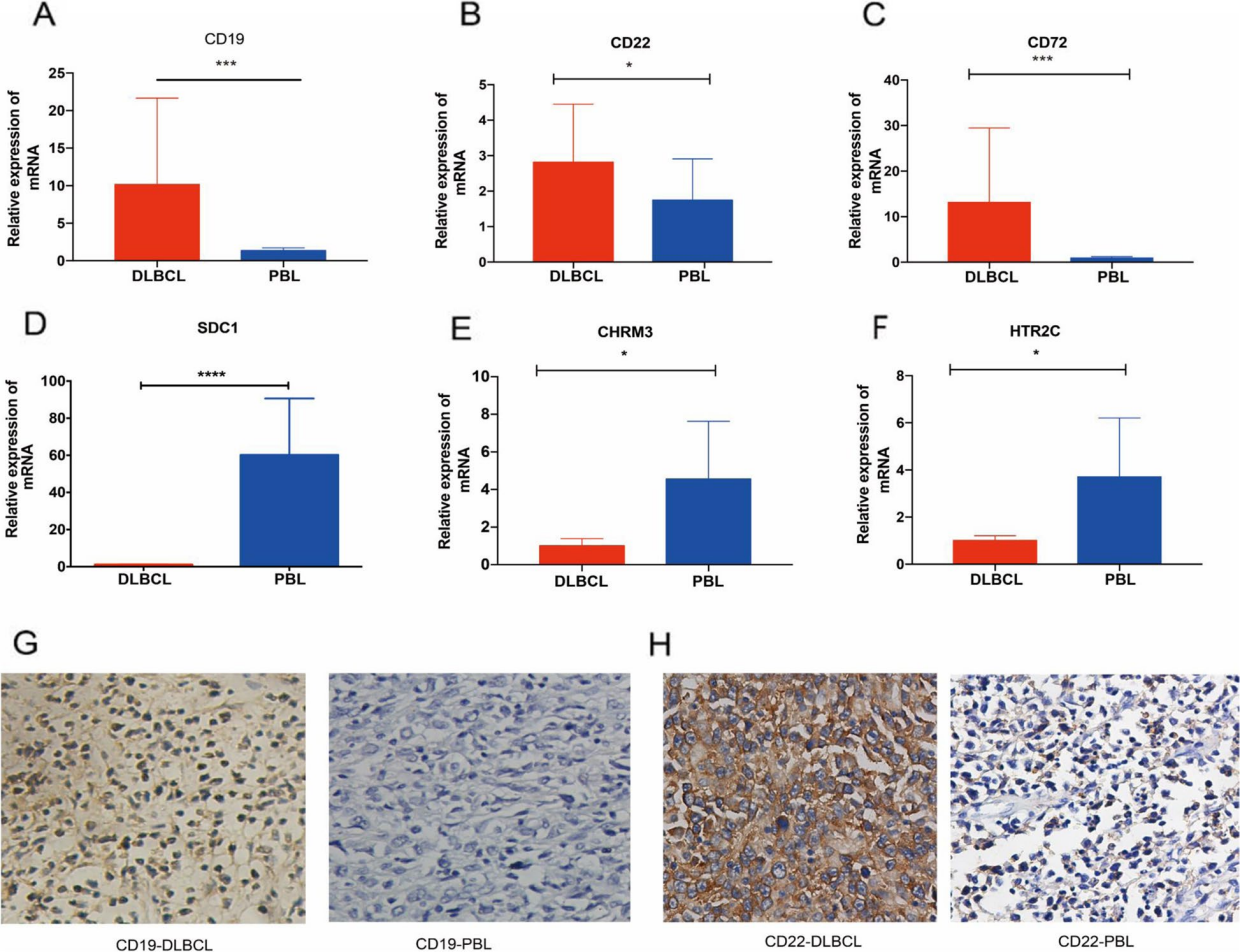
Validation of DEGs by RT-qPCR and immunohistochemistry. **A**–**C** The qRT-PCR results showed that the mRNA expression of CD19, CD22, and CD72 were relatively lower in PBL than in DLBCL. **D**–**F** The RT-qPCR results showed that the expression of SDC1, CHRM3, and HTR2C were relatively higher in PBL were relatively lower in PBL than in DLBCL. **G**, **H** The expression of CD19 and CD22 detected by IHC. Magnification, x200

To further confirm the results, RT-qPCR analysis and IHC were performed to validate the DEGs from the RNA-seq data. Gene expression data were selectively validated by real-time PCR for some candidate genes, which were chosen based on the differential fold change between DLBCLs and PBLs. RT-qPCR results showed that the mRNA expression of CD19 (BCR signaling pathway), CD22 (BCR signaling pathway), and CD72 (BCR signaling pathway) was relatively lower in PBL than in DLBCL (Fig. 4A–C), while the expression of SDC1 (Cell adhesion molecule), CHRM3 (calcium signaling pathway), and HTR2C (calcium signaling pathway) was relatively higher in PBL than in DLBCL (Fig. 4D–F). In addition, IHC result demonstrated that the expression of BCR signaling genes (CD19 and CD22) were relatively lower in PBL compared to DLBCL (Fig. 4G, H). The RT-qPCR and IHC results highly correlated with the array data, indicating that the RNA-seq data was reliable and accurate.

## Discussion

PBL is a rare type of non-Hodgkin lymphoma with overlapping features of B-cell lymphoma and plasma cell neoplasms [11]. It is characterized by male predominance, frequent involvement of the oral cavity, and an aggressive disease course, with a high rate of HIV and EBV infection [1, 3]. The exact pathogenesis, standard treatment strategies, and prognostic factors have not yet been defined. This study examined the clinicopathological characteristics, molecular features, and clinical outcomes of PBL in Chinese population.

We found that the clinicopathologic features, such as patient immune status, lesion location, and clinical outcome, were different between Chinese and Western populations. Many studies have confirmed that PBL mainly affects immunocompromised individuals, such as those with HIV infection or other autoimmune diseases [1–3, 8, 11]. Patients in our study group were HIV-negative, and most cases were immunocompetent, consistent with the findings of a review of 60 Chinese PBL patients [12]. In addition, the most involved sites in our study were the lymph nodes and gastrointestinal tract, followed by the oral cavity, which differed from the findings of previous research that reported that PBL commonly involved the oral cavity and other extra nodal sites in patients with immunodeficiency in Europe [1, 3, 8]. Similar to previous findings [1, 3, 7], plasma cell markers (CD38, CD138, and MUM1) were commonly expressed in our patients.

There is no optimal therapeutic approach for PBL. Although CHOP was the most commonly used regimen and patients achieved an overall response rate of approximately 60–70% after treatment, the NCCN guidelines demonstrated that it is inadequate therapy and recommends a more intensive regimen, including hyper-CVAD, CODOX-M/IVAC, and DA-EPOCH [13]. Previous studies have shown no apparent survival benefit of intensive chemotherapy over CHOP regimens in PBL patients [14, 15]. The prognosis of PBL remains poor, with a median OS of 6–32 months [1, 7, 8]. However, the survival outcome of our cohort was relatively longer, with 2-year PFS and OS of 62.7% and 65.2%, respectively. More than half of the patients in our cohort were at an early stage of disease and this may partly explain the prognosis.

Some important prognostic factors, such as disease stage, serum LDH level, EBV status, and CR status, have been identified in PBL [1, 2]. In our study, clinical stage, LDH level, EBV status, patient CR status, and IPI were significant prognostic factors in the univariate analysis, consistent with the findings of other studies [1, 2]. However, only CR status remained a significant independent factor in the multivariate analysis [1]. Many studies have reported that CR after treatment is associated with better outcomes in PBL patients, suggesting that CR is one of the strongest prognostic factors.

The chronic activation of the B cell receptor (BCR) and various downstream signals has been reported to be important for the survival of B cell lymphoma [16, 17]. In recent years, BCR signaling has emerged as an established target in lymphoma, and BCR inhibitors have achieved clinical effects in B-cell lymphoma [18, 19]. In our study, BCR signal was downregulated in PBL compared to DLBCL, which was consistent with the findings of a previous study [20], indicating that the BCR signal was not central to the pathogenesis of PBL and that PBL was distinct from DLBCL.

The tumor suppressor gene *TP53*, which encodes the p53 transcription factor and then regulates many target genes in various cancers, was reported to be a barrier to tumor development [21–25]. Mutations in *TP53* occurred in around 20% of DLBCLs and loss of P53 function could contribute to lymphomagenesis [25, 26]. In ABC-DLBCL, loss of P53 function could facilitate tumor progression by suppressing the pathogenic cooperation of IKK2ca-enforced canonical NF-kB [24]. Our results showed that P53 signaling pathway was significantly downregulated in PBL compared to GCB-DLBCL, indicating that inactivated P53 may contribute to lymphomagenesis in PBL and serve as a potential therapeutic target in the future.

The tumor microenvironment can interact with cancer cells and play a critical role in tumor development and drug resistance [27, 28]. Cell adhesion molecules (CAMs) can mediate interactions between tumor cells and stromal cells. Recent studies have reported that high expression of CAMs contributes to the activation of multiple signaling pathways and promotes the development of cancer in plasma cell neoplasms and lymphoma [27–29]. Targeting CAMs such as CD38 and CD138/SDC1 with monoclonal antibodies have achieved promising results in multiple myeloma [30, 31]. Daratumumab, a monoclonal antibody directed against CD38, was reported to be effective in advanced-stage large B-cell lymphoma (LBCL) with plasmablastic features [32]. In this study, four PBL patients achieved durable response (12–31 months and ongoing) after the treatment of daratumumab combined with DA-EPOCH [32]. Our results showed that the expression of CAMs was significantly higher in PBL than in DLBCL, suggesting that CAMs may play important roles in the development of PBL. In addition, CAMs may be potential therapeutic targets in PBL and require further investigation in the future.

## Conclusions

The findings of our study demonstrated a higher frequency of primary extra nodal involvement and indicated that HIV status was different in Chinese PBL patients than that commonly underscored in the literature. Patients with early-stage disease and CR after treatment may have a favorable prognosis. BCR signaling was downregulated in PBL patients compared to DLBCL patients, indicating that this signaling may play a small role in PBL. The downregulated P53 signaling pathway may contribute to the lymphomagenesis in PBL and serve as a potential therapeutic target in the future. In addition, significant upregulation of cell adhesion genes was identified in PBL, and these CAMs may be potential therapeutic targets in the future. This study has several limitations. This study was retrospective and lacked a comparator group. Another shortcoming was that the treatment and follow-up data was missing in approximately half of the patients and may lead to a significant ascertainment bias in the outcome result. In addition, the sample size for RNA-sequencing was relatively small in our study. Additional large-scale, prospective, and international studies are needed to further identify the concrete pathogenesis and molecular features of PBL and improve the survival outcome of PBL.

## Supplementary Information

**Additional file1. Table S1**: RT-qPCR primers.** Table S2**: Clinicopathologic characteristics of PBL patients in our study. **Table S3**: Clinicopathologic characteristics of PBL patients and DLBCL patients for RNA sequencing in our study. **Fig. S1**: Expression profiles. A. Hierarchical clustering and heat map of DEGs between PBL and GCB-DLBCL. Red blocks represent the overexpressed genes, while the blue blocks represent the lowly expressing genes. B. Volcano plot of DEGs (FDR < 0.05) between PBL and GCB-DLBCL. C. Significantly upregulated pathways in PBL compared to GCB-DLBCL. D. Significantly downregulated pathways in PBL compared to GCB-DLBCL. -F. Downregulation of DEGs of BCR E and P53 F signaling pathways in PBLs compared to GCB-DLBCL. **Fig. S2**: Expression profiles. A. Hierarchical clustering and heat map of DEGs between PBL and non-GCB-DLBCL. Red blocks represent the overexpressed genes, while the blue blocks represent the lowly expressing genes. B. Volcano plot of DEGs (FDR < 0.05) between PBL and non-GCB-DLBCL. C. Significantly upregulated pathways in PBL compared to non-GCB-DLBCL. D. Significantly downregulated pathways in PBL compared to GCB-DLBCL. E-H. Downregulation of DEGs of BCR E, TCR F, Jak-STAT G and TLR H signaling pathways in PBLs compared to GCB-DLBCL.

## Data Availability

The datasets used and/or analyzed in this study are available from the corresponding author upon reasonable request.

## Abbreviations

BCR: B cell receptor
CAMs: Cell adhesion molecules
CR: Complete remission
DEGs: Differentially expressed genes
DLBCL: Diffuse large B-cell lymphoma
EBER: EBV-encoded small RNA
EBV: Epstein-Barr virus
GDC: Genomic Data Commons
HIV: Human immunodeficiency virus
IPI: International prognostic index
LDH: Lactate dehydrogenase
PBL: Plasmablastic lymphoma
PCM: Plasma cell myeloma
PFS: Progression-free survival
ORR: Overall response rate
OS: Overall survival
RT-qPCR: Reverse-transcriptase quantitative PCR
