# Supplementary material for: Clinicopathologic features and abnormal signaling pathways in plasmablastic lymphoma: a multicenter study in China
Source: BMC Med. 2022 Dec 15;20:483. doi: 10.1186/s12916-022-02683-9 (PMC9753322; doi:10.1186/s12916-022-02683-9)
Supplement: Supplementary file 1 — Additional file1. Table S1: RT-qPCR primers. Table S2: Clinicopathologic characteristics of PBL patients in our study. Table S3: Clinicopathologic characteristics of PBL patients and DLBCL patients for RNA sequencing in our study. Fig. S1: Expression profiles. A. Hierarchical clustering and heat map of DEGs between PBL and GCB-DLBCL. Red blocks represent the overexpressed genes, while the blue blocks represent the lowly expressing genes. B. Volcano plot of DEGs (FDR < 0.05) between PBL and GCB-DLBCL. C. Significantly upregulated pathways in PBL compared to GCB-DLBCL. D. Significantly downregulated pathways in PBL compared to GCB-DLBCL. -F. Downregulation of DEGs of BCR E and P53 F signaling pathways in PBLs compared to GCB-DLBCL. Fig. S2: Expression profiles. A. Hierarchical clustering and heat map of DEGs between PBL and non-GCB-DLBCL. Red blocks represent the overexpressed genes, while the blue blocks represent the lowly expressing genes. B. Volcano plot of DEGs (FDR < 0.05) between PBL and non-GCB-DLBCL. C. Significantly upregulated pathways in PBL compared to non-GCB-DLBCL. D. Significantly downregulated pathways in PBL compared to GCB-DLBCL. E-H. Downregulation of DEGs of BCR E, TCR F, Jak-STAT G and TLR H signaling pathways in PBLs compared to GCB-DLBCL. [file 12916_2022_2683_MOESM1_ESM.pdf]

Table S1. RT-PCR primers

GENE	Sequence
SDC1-F	5’-CTGCCGCAAATTGTGGCTAC-3’
SDC1-R	5’-TGAGCCGGAGAAGTTGTCAGA-3’
CHRM3-F	5’-CACAATAACAGTACAACCTCGCC-3’
CHRM3-R	5’-GCCAGGATGCCCGTTAAGAAA-3’
HTR2C-F	5’-CTAATTGGCCTATTGGTTTGGCA-3’
HTR2C-R	5’-CCACCATCGGAGGTATTGAAAA-3’
CD22-F	5’-GCACCCTGAAACCCTCTACG-3’
CD22-R	5’-ATCAAACTTCGAGGTGTTCTTGT-3’
CD72-F	5’-ATCTGAGGTTTGTGAAGGCTCC-3’
CD72-R	5’-AACATTCTCGTAGGTGATTTCCC-3’
CD19-F	5’-CCCAAGGGGCCTAAGTCATTG-3’
CD19-R	5’-AACAGACCCGTCTCCATTACC-3’
GAPDH-F	5’-TGCACCACCAACTGCTTAGC-3’
GAPDH-R	5’-GGCATGGACTGTGGTCATGAG-3’

Table S2. Clinicopathologic characteristics of PBL patients in our study

Variable	Total N (%)
Patients	102
Age (years)	
>60	39(38.2)
≤60	63(61.8)
Median (years)	55
Sex	
Male	68(66.7)
Female	34(33.3)
Site(s) of involvement	
Lymph involvement	24(23.5)
Gastro-intestinal tractus	20(19.6)
Oral cavity	16(15.7)
bone	12(11.8)
skin	7(6.9)
Ear, nose, throat site	6(5.9)
lung	5(4.9)
Maxillary sinus	3(2.9)
peritoneum	3(2.9)
liver	2(2.0)
adrenal gland	2(1.9)
uterus	1(1.0)
Soft tissue	1(1.0)
Immunohistochemistry	
CD38	49/55 (89.1)
CD138	54/79 (68.3)
MUM1	77/86 (89.5)
EBER	15/36
Ki67 (median)	80.0%

Table S3. Clinicopathologic characteristics of PBL patients and DLBCL patients for RNA sequencing in our study

Variable	DLBCL	PBL	*P* value
Patients	11	6	
Age (years)			>0.99
>60	5	3	
≤60	6	3	
Sex			>0.99
Male	8	5	
Female	3	1	
Primary site(s)			>0.99
Lymph node	1	1	
Extranodal sites	10	5	
Ann Arbor stage			>0.99
I or II	7	4	
III or IV	4	2	

Figure S1

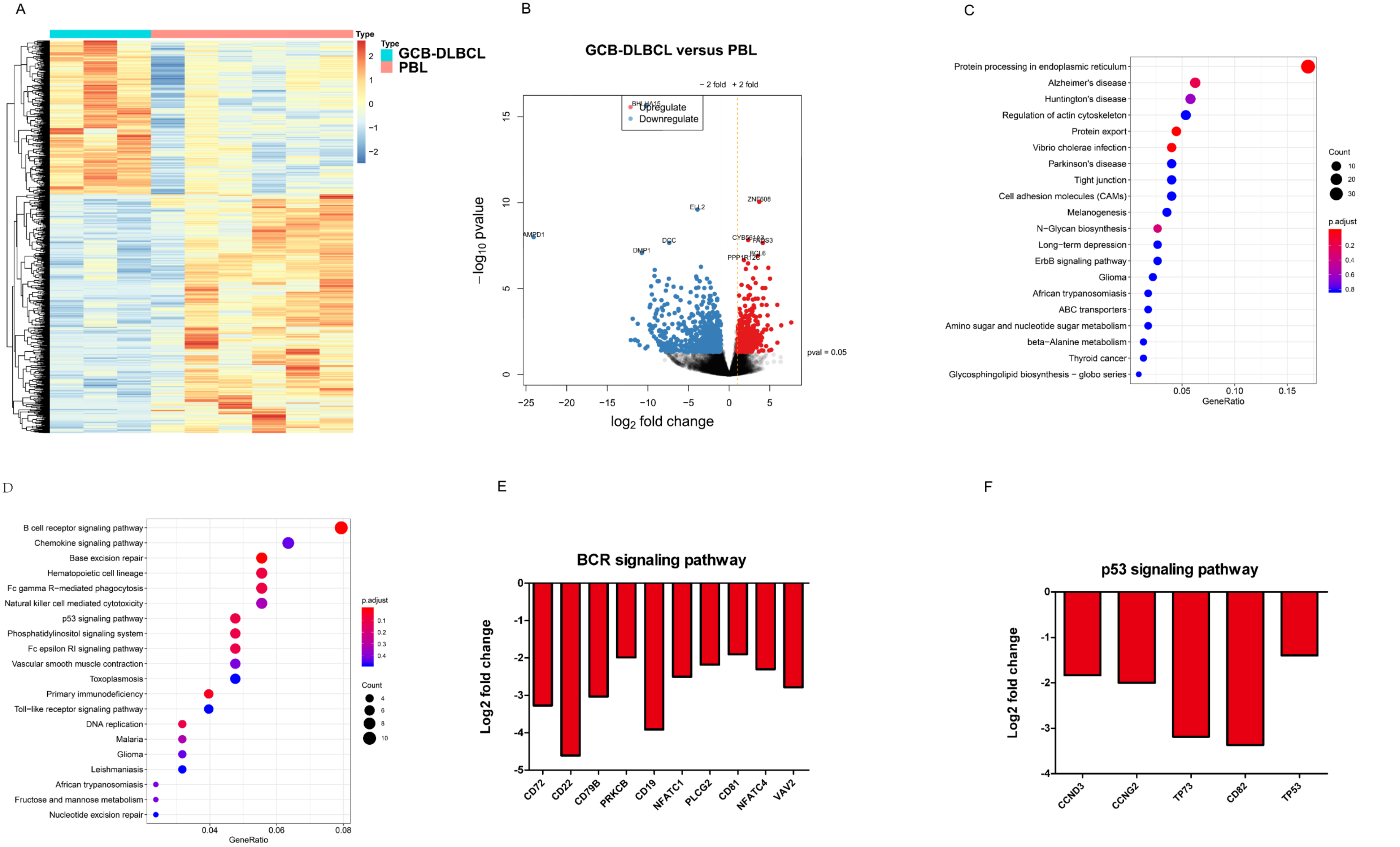
A
Type
GCB-DLBCL
PBL
B
GCB-DLBCL versus PBL
Upregulate
Downregulate
− 2 fold + 2 fold
pval = 0.05
−log10 pvalue
log2 fold change
C
Protein processing in endoplasmic reticulum
Alzheimer's disease
Huntington's disease
Regulation of actin cytoskeleton
Protein export
Vibrio cholerae infection
Parkinson's disease
Tight junction
Cell adhesion molecules (CAMs)
Melanogenesis
N−Glycan biosynthesis
Long−term depression
ErbB signaling pathway
Glioma
African trypanosomiasis
ABC transporters
Amino sugar and nucleotide sugar metabolism
beta−Alanine metabolism
Thyroid cancer
Glycosphingolipid biosynthesis − globo series
Count
p.adjust
GeneRatio
D
B cell receptor signaling pathway
Chemokine signaling pathway
Base excision repair
Hematopoietic cell lineage
Fc gamma R−mediated phagocytosis
Natural killer cell mediated cytotoxicity
p53 signaling pathway
Phosphatidylinositol signaling system
Fc epsilon RI signaling pathway
Vascular smooth muscle contraction
Toxoplasmosis
Primary immunodeficiency
Toll−like receptor signaling pathway
DNA replication
Malaria
Glioma
Leishmaniasis
African trypanosomiasis
Fructose and mannose metabolism
Nucleotide excision repair
p.adjust
Count
GeneRatio
E
BCR signaling pathway
Log2 fold change
CD72
CD22
CD79B
PRKCB
CD19
NFATC1
PLCG2
CD81
NFATC4
VAV2
F
p53 signaling pathway
Log2 fold change
CCND3
CCNG2
TP73
CD82
TP53

Figure S2

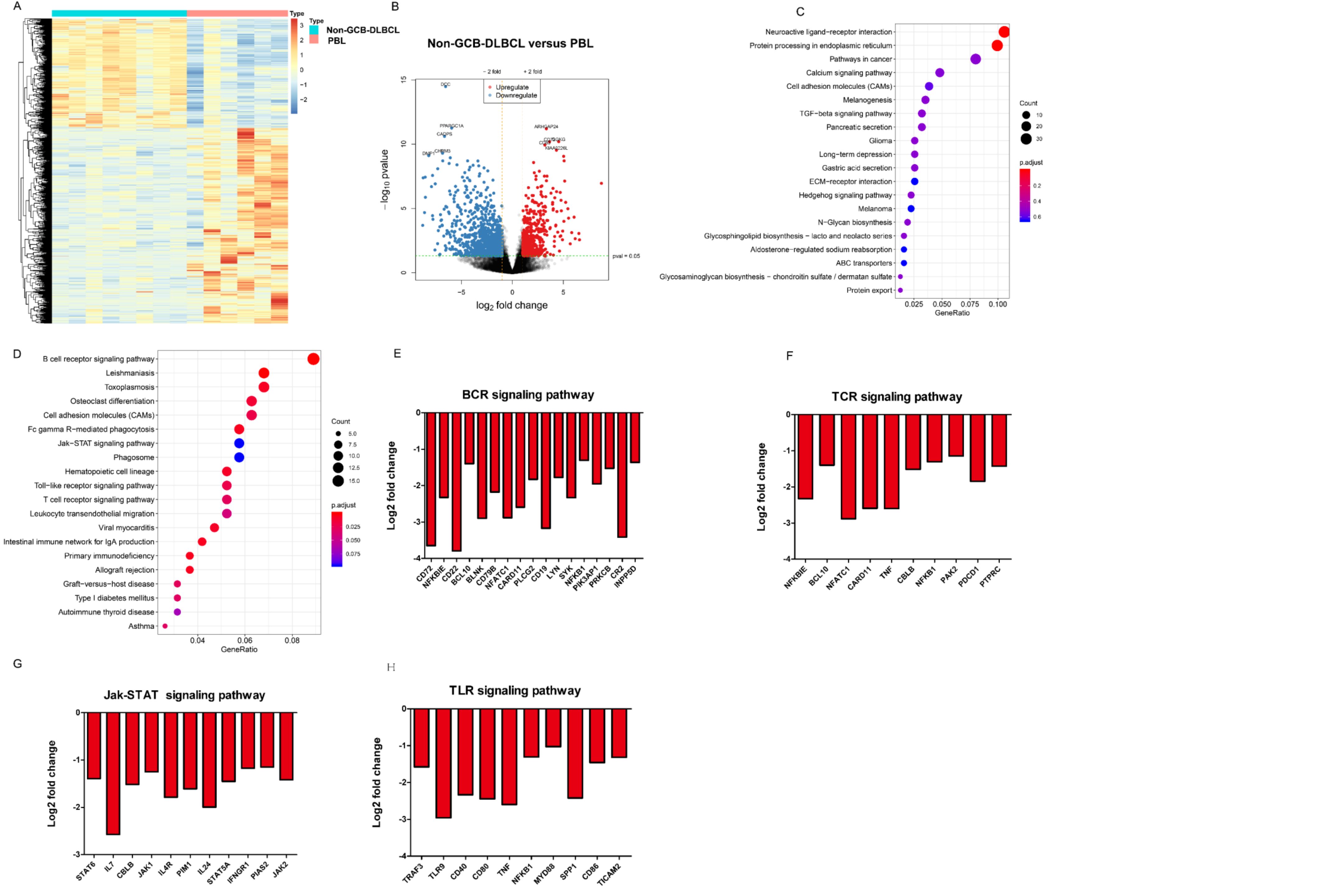

A
Type
Non-GCB-DLBCL
PBL
B
Non-GCB-DLBCL versus PBL
− 2 fold
+ 2 fold
Upregulate
Downregulate
pval = 0.05
−log10 pvalue
log2 fold change
C
Neuroactive ligand−receptor interaction
Protein processing in endoplasmic reticulum
Pathways in cancer
Calcium signaling pathway
Cell adhesion molecules (CAMs)
Melanogenesis
TGF−beta signaling pathway
Pancreatic secretion
Glioma
Long−term depression
Gastric acid secretion
ECM−receptor interaction
Hedgehog signaling pathway
Melanoma
N−Glycan biosynthesis
Glycosphingolipid biosynthesis − lacto and neolacto series
Aldosterone−regulated sodium reabsorption
ABC transporters
Glycosaminoglycan biosynthesis − chondroitin sulfate / dermatan sulfate
Protein export
Count
p.adjust
GeneRatio
D
B cell receptor signaling pathway
Leishmaniasis
Toxoplasmosis
Osteoclast differentiation
Cell adhesion molecules (CAMs)
Fc gamma R−mediated phagocytosis
Jak−STAT signaling pathway
Phagosome
Hematopoietic cell lineage
Toll−like receptor signaling pathway
T cell receptor signaling pathway
Leukocyte transendothelial migration
Viral myocarditis
Intestinal immune network for IgA production
Primary immunodeficiency
Allograft rejection
Graft−versus−host disease
Type I diabetes mellitus
Autoimmune thyroid disease
Asthma
Count
p.adjust
GeneRatio
E
BCR signaling pathway
Log2 fold change
CD72 NFKBIE CD22 BCL10 BLNK CD79B NFATC1 CARD11 PLCG2 CD19 LYN SYK NFKB1 PIK3AP1 PRKCB CR2 INPP5D
F
TCR signaling pathway
Log2 fold change
NFKBIE BCL10 NFATC1 CARD11 TNF CBLB NFKB1 PAK2 PDCD1 PTPRC
G
Jak-STAT signaling pathway
Log2 fold change
STAT6 IL7 CBLB JAK1 IL4R PIM1 IL24 STAT5A IFNGR1 PIAS2 JAK2
H
TLR signaling pathway
Log2 fold change
TRAF3 TLR9 CD40 CD80 TNF NFKB1 MYD88 SPP1 CD86 TICAM2
